# Supplementary material for: An open-source framework for large-scale, flexible evaluation of biomedical text mining systems
Source: J Biomed Discov Collab. 2008 Jan 29;3:1. doi: 10.1186/1747-5333-3-1 (PMC2276192; doi:10.1186/1747-5333-3-1)
Supplement: Additional file 1 — Evaluation platform source code. The additional file contains the software evaluation framework discussed in this paper. The most current release can be downloaded from SourceForge.net [1]. See the accompanying README file for instructions on installation and use. Also included in the distribution is a collection of UIMA wrappers for some commonly used BioNLP tools and annotated corpora. [file 1747-5333-3-1-S1.zip › bionlp_uima_repository-1.0.1/build/test/reports/unit/edu/uchsc/ccp/util/nlp/annotation/comparison/AnnotationComparatorTest.html]

Unit Test Results: edu.uchsc.ccp.util.nlp.annotation.comparison.AnnotationComparatorTest


# Unit Test Results

|  |  |
| --- | --- |
|  | Designed for use with JUnit and Ant. |

---

### Class edu.uchsc.ccp.util.nlp.annotation.comparison.AnnotationComparatorTest

| Name | Tests | Errors | Failures | Time(s) |
| --- | --- | --- | --- | --- |
| AnnotationComparatorTest | 13 | 0 | 0 | 2.343 |

## Tests

| Name | Status | Type | Time(s) |
| --- | --- | --- | --- |
| testDefaultCompare | Success |  | 0.020 |
| testDefaultCompareLists | Success |  | 0.399 |
| testCompareWithStrictSpanIdenticalMention | Success |  | 0.001 |
| testCompareListsWithStrictSpanIdenticalMention | Success |  | 0.309 |
| testCompareWithSloppySpanIdenticalMention | Success |  | 0.001 |
| testCompareListsWithSloppySpanIdenticalMention | Success |  | 0.331 |
| testCompareWithSharedStartSpanIdenticalMention | Success |  | 0.001 |
| testCompareListsWithSharedStartSpanIdenticalMention | Success |  | 0.287 |
| testCompareWithSharedEndSpanIdenticalMention | Success |  | 0.001 |
| testCompareListsWithSharedEndSpanIdenticalMention | Success |  | 0.281 |
| testCompareWithSharedEitherSpanIdenticalMention | Success |  | 0.005 |
| testCompareListsWithSharedEitherSpanIdenticalMention | Success |  | 0.284 |
| testEntityPartsAnnotationComparator | Success |  | 0.417 |

Properties »
